# Optical metrics of the extracellular matrix predict compositional and mechanical changes after myocardial infarction

**DOI:** 10.1038/srep35823

**Published:** 2016-11-07

**Authors:** Kyle P. Quinn, Kelly E. Sullivan, Zhiyi Liu, Zachary Ballard, Christos Siokatas, Irene Georgakoudi, Lauren D. Black

**Affiliations:** 1 Department of Biomedical Engineering, Tufts University, Medford, MA, 02155, USA; 2 Department of Biomedical Engineering, University of Arkansas, Fayetteville, AR, 72701, USA; 3 Department of Chemistry, Aristotle University of Thessaloniki, Thessaloniki, 54124, Greece; 4 Cellular, Molecular, and Developmental Biology Program, Sackler School for Graduate Biomedical Sciences, Tufts University School of Medicine, Boston, MA 02111, USA

## Abstract

Understanding the organization and mechanical function of the extracellular matrix (ECM) is critical for the development of therapeutic strategies that regulate wound healing following disease or injury. However, these relationships are challenging to elucidate during remodeling following myocardial infarction (MI) due to rapid changes in cellularity and an inability to characterize both ECM microstructure and function non-destructively. In this study, we overcome those challenges through whole organ decellularization and non-linear optical microscopy to directly relate the microstructure and mechanical properties of myocardial ECM. We non-destructively quantify collagen organization, content, and cross-linking within decellularized healthy and infarcted myocardium using second harmonic generation (SHG) and two photon excited autofluorescence. Tensile mechanical testing and compositional analysis reveal that the cumulative SHG intensity within each image volume and the average collagen autofluorescence are significantly correlated with collagen content and elastic modulus of the ECM, respectively. Compared to healthy ECM, infarcted tissues demonstrate a significant increase in collagen content and fiber alignment, and a decrease in cross-linking and elastic modulus. These findings indicate that cross-linking plays a key role in stiffness at the collagen fiber level following infarction, and highlight how this non-destructive approach to assessing remodeling can be used to understand ECM structure-function relationships.

Extracellular matrix (ECM) remodeling following myocardial infarction (MI) is critical for stabilizing the injured myocardium to prevent aneurysmal deformation^1^,^2^. Following a significant ischemic event, and in conjunction with a massive inflammatory response, cardiac fibroblasts replace the necrotic myocardium with a fibrotic scar, which provides the structural support required to withstand the dynamic forces present during ventricular filling and contraction^3^. The deposition of new collagen matrix often starts within the first week^4^ and steadily increases until a maximum is reached 3 to 6 weeks post-infarction^5^. However, myocardial remodeling can often be excessive, marked by the expansion of the scar, thinning of the ventricular wall, and remodeling of the ECM within the remote, non-infarcted zone. A transition from healing to adverse remodeling occurs as the shape, mass, and volume of the left ventricle changes^6^. With time, functionality of the left ventricle continues to decline and eventually contributes to the progression towards heart failure. Alterations in the mechanical properties of the myocardium resulting from matrix remodeling are often responsible for functional deterioration. Specifically, the increased myocardial stiffness observed starting 1 to 3 weeks following MI^7^ decreases ventricular compliance^8^ and impairs cardiomyocyte relaxation^9^, which leads to diastolic dysfunction and contributes to heart failure progression^10^.

Several characteristics of the ECM influence its mechanical function, including collagen content^11^, collagen type^12^, fiber diameter^13^, fiber orientation^14^ and cross-linking^15^,^16^. However, their relative contributions to the mechanical response of the ECM have yet to be clearly defined^17^, particularly in the context of MI. One of the most thorough investigations of structure-function relationships of the myocardium following infarction utilized planar biaxial testing, collagen assays, and polarized light microscopy of histological sections and concluded that collagen fiber diameter and content are the main determinants of scar stiffness in a rat model^7^. However, the relationship between the ECM composition of the scar and its mechanical properties during the acute stages of remodeling is challenging to define because of rapid changes in the cellular populations and morphology of the heart following infarction^18^.

In the healthy adult heart, contractile cardiomyocytes comprise nearly 75% of the myocardial volume^19^. These cells quickly become necrotic and apoptotic following ischemic injury and their cellular debris constitutes a significant portion of the scar volume but contributes minimally to passive tissue stiffness. This substantial decrease in cellularity in conjunction with matrix metalloproteinase-mediated ECM remodeling likely contribute to the reduced structural integrity of the myocardium during the acute stages of infarct remodeling^20^. Although tissue strength is reduced during the early stages of remodeling, many studies have also reported an increase in the elastic modulus by 3 weeks post-MI^7^,^21^. Yet it remains unclear whether this increase in the overall tissue stiffness is driven by a change in the elastic moduli of the cells and/or ECM, or simply a change in the relative volume occupied by cells and ECM proteins. It is established that the relative volume fraction does change as new collagen matrix is deposited and necrotic cardiomyocytes are removed by inflammatory mediators^22^, but the specific mechanical properties of the ECM may also change in the weeks following MI. To elucidate the structure-function relationships of the ECM in the absence of the changing cellular constituents of the heart, whole organ perfusion decellularization can be utilized to isolate and specifically characterize the ECM^23^.

A number of key insights into myocardial structure-function relationships have been obtained by combining mechanical testing with histology. However, the tissue processing steps that are used in histology are destructive and can potentially introduce artifacts in the perceived tissue microstructural organization during fixation, sectioning, and staining. Therefore, non-destructive imaging approaches that quantify the content and three dimensional organization of intact collagenous tissues, such as non-linear optical microscopy, can improve the characterization of tissue microstructure^24^,^25^. Because collagen molecules have a non-centrosymmetric structure, they are capable of producing a strong second harmonic generation (SHG) signal, which has previously been utilized to assess collagen remodeling^26^,^27^,^28^. For decades, spectroscopic studies have also reported collagen autofluorescence emission in the ultraviolet and visible range that can largely be attributed to their cross-links^29^,^30^. Yet, few studies have explored the two-photon excited fluorescence (TPEF) of collagen cross-links during simultaneous SHG imaging^24^,^30^,^31^. Given that collagen cross-links may play a key role in determining the mechanical properties of the myocardium^15^,^32^, the objective of this study was to utilize TPEF and SHG imaging to characterize collagen fiber organization and cross-linking status following MI in a rat model and elucidate the relationship between tissue microstructure and the mechanical properties of the ECM. Due to the non-destructive nature of 3D multi-photon microscopy, direct paired comparisons could be made between quantitative optical imaging outcomes, traditional mechanical testing, and compositional assays of ECM content. This integrative analysis of ECM structure, content, and mechanical function provided a direct measure of how collagen cross-linking plays a key role in determining the mechanical properties of the ECM during scar remodeling. Here we demonstrate how the deposition of new collagen fibers after MI results in an ECM with inferior material properties. Further application of this methodology will be valuable for the evaluation of therapeutic strategies which aim to manipulate ECM remodeling following MI.

## Methods

### Animal model

An expanded Methods section is available online as Supplementary Material. Animal experiments were conducted in accordance with Tufts University guidelines, NIH guidelines, and the US Animal Welfare Act and approved by the Tufts Institutional Animal Care and Use Committee, protocol M2014–31. Male Sprague-Dawley rats (2+ months, 250–275 grams) were anesthetized with isoflurane (4% induction, 2% maintenance) for MI induction and echocardiography. Anesthetic depth was monitored by respiratory rate and toe pinch. The analgesic Bupivicaine was delivered (2 mg/kg subcutaneously) in the intercostal region prior to surgery, while Buprenorphine was administered (0.05 mg/kg subcutaneously) post-operatively and repeated every 12 hours for the next 72 hours. Induction of a significant infarction was verified if 40–50% of the left ventricle blanched following coronary artery ligation. Long and short axis echocardiograms were collected prior to sacrifice at 4 and 8 weeks post-MI for the estimation of ejection fraction using Simpson’s 2D biplane methodology^33^. Rats were euthanized by CO_2_ asphyxiation and a thoracotomy prior to MI and 1, 2, 4, and 8 weeks post-MI. The harvested hearts were decellularized via retrograde perfusion with 1% SDS, which was confirmed with a DNA assay^23^. The infarcted region of the heart was excised and sectioned into three circumferential strips of similar width (between 2–4 mm) and classified as base, mid, or apex regions of the scar. All decellularized samples were characterized through multiphoton microscopy followed by uniaxial mechanical testing and finally assessed for hydroxyproline content (n = 4 or more animals/condition). Two additional subsets of hearts were acquired for analysis via H&E staining (n = 1 animal/condition) and LC-MS/MS proteomics (n = 3 animals/condition). While samples were decellularized for LC-MS/MS proteomics analysis, native tissue samples were collected for cryosectioning and staining. A total of 79 animals were used for this study.

### Multiphoton microscopy

Images of the decellularized tissue samples were obtained with a Leica TCS SP2 confocal microscope equipped with a titanium-sapphire laser (Mai Tai; Spectra Physics; Mountain View, CA) and a 63× objective (NA 1.2). Images (512 × 512 pixels) were acquired at 5 μm z-steps by two non-descanned PMT detectors with 400(±10) nm and 525(±25) nm emission filters using 740 nm and 800 nm excitation. Image intensities were normalized by PMT gain and laser power as previously described^34^. A mask of collagen-positive voxels was defined where the filtered SHG intensity exceeded 0.05. The average intensity within the collagen mask was computed for each of the 4 image channels (SHG, TPEF 740/400, TPEF 740/525, TPEF 800/525). The cumulative collagen autofluorescence and SHG intensity within each image volume were also computed by summing all intensity values within the collagen voxels and normalizing by the total image volume. Fiber orientation was determined through 2-D Fourier transform and angular sampling; mean fiber orientation and directional variance were computed from the derived angular distribution.

### Mechanical and compositional analysis

Tissues were loaded in tension in the circumferential direction of the heart using a custom-built uniaxial mechanical testing machine. Stress-stretch data were analyzed using a probabilistic model of fiber recruitment described by a Weibull distribution that has previously demonstrated an ability to model the behavior of different soft, connective tissues^35^. The shape parameter, β, of the Weibull distribution was fixed to 4. The stretch value where the first fibers contribute stiffness (γ), scale of the stretch values over which recruitment occurs (δ), and elastic modulus (E) following total fiber recruitment were determined through curve fitting in Matlab. After mechanical testing, composition of the infarcted myocardium was characterized through a QuickZyme total collagen assay. Please see additional details regarding the mechanical and compositional analysis in the Supplementary Material.

### Statistical Analysis

Two-way ANOVAs with scar region (base, mid, apex) and time point (healthy, weeks 1, 2, 4, 8 post-MI) effects were used to assess significant differences for each optical, hydroxyproline, and mechanical metric. One-way ANOVAs with time point (healthy, weeks 1, 2, 4, 8 post-MI) effects were used to assess significant differences in all other data sets. In both cases, post hoc Tukey tests were used to determine differences among specific time points. Correlation coefficients between optical, compositional, and mechanical metrics were determined significant based on the null hypothesis that R = 0. Significance was defined by α = 0.05.

## Results

**Figure 1:**
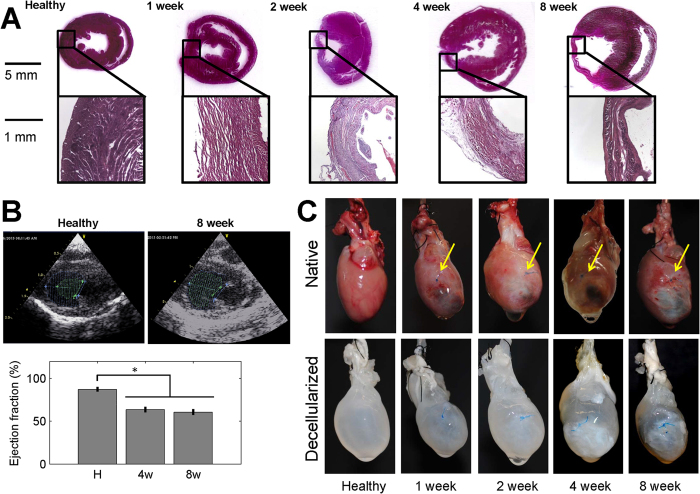
Characterization of rat model of myocardial infarction. (**A**) Ventricular free wall thinning occurs in the region of the infarct as demonstrated by hematoxylin and eosin staining of native heart sections (n = 1 per time point). (**B**) Representative long axis echocardiograms of healthy and 8 week infarct hearts demonstrate a loss of contractility following MI. End diastolic volume (purple, dashed line) and end systolic volume (green, dashed line) were estimated in order to calculate ejection fraction according to Simpson’s 2D biplane methodology. A significant reduction in ejection fraction was identified at 4 and 8 weeks post-MI relative to healthy (One-way ANOVA identified p < 0.001, n = 4–5 per group). (**C**) Isolated healthy and infarcted hearts before and after decellularization (n = 1 per time point). Yellow arrows highlight where the coronary artery was ligated. Size and density of scar appears to increase with time post-MI.

**Figure 2:**
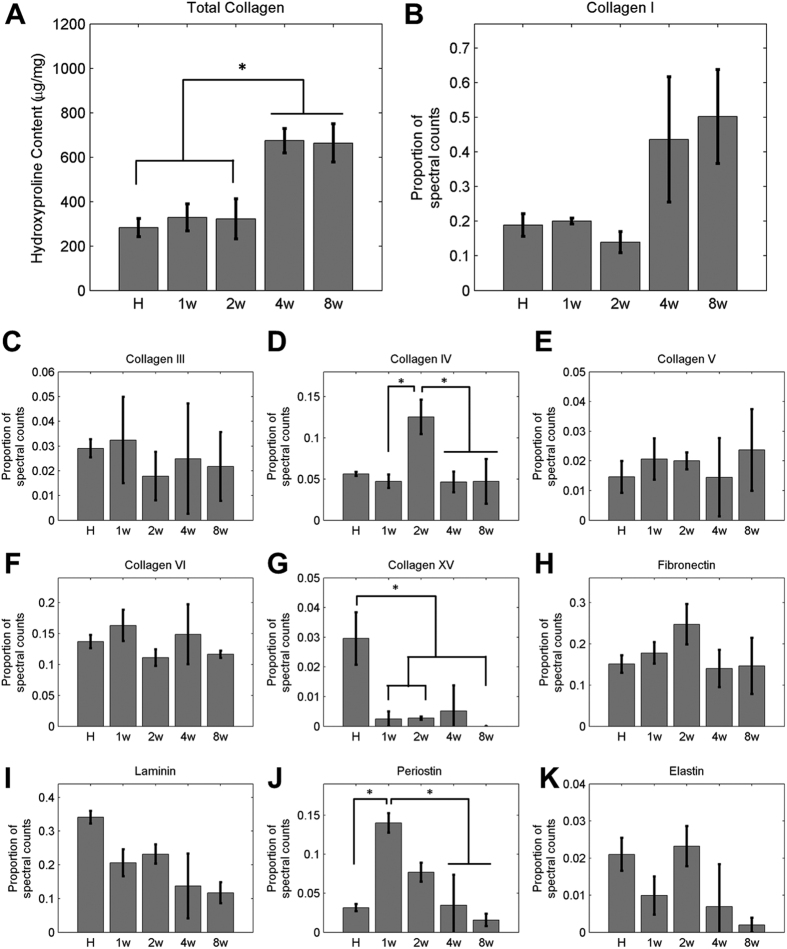
Composition of decellularized myocardial matrix varies with time post-MI. (**A**) A hydroxyproline assay demonstrated a significant increase in total collagen at 4 and 8 week time points relative to healthy and 2 weeks (n = 5–8/time point). Spectral count analysis from LC-MS/MS of decellularized myocardium identified a similar trend in Collagen I content (**B**) with increased abundance at 4 and 8 weeks. (**C**) Collagen III content remained relatively stable throughout the remodeling cascade. (**D**) Collagen IV content within the myocardial matrix was most abundant at 2 weeks post-MI relative to 1, 4 and 8 week time points. The abundance of (**E**) Collagen V and (**F**) Collagen VI within the decellularized matrix was not altered as a function of remodeling time, while (**G**) Collagen XV was significantly more abundant within the healthy samples as compared to the 1, 2 or 8 week conditions. (**H**) Fibronectin demonstrated a trend towards increasing abundance at 2 weeks post-MI, but did not reach statistical significance. (**I**) The proportion of Laminin within the spectra decreased following MI, while the abundance of (**J**) Periostin increased dramatically at 1 week post-MI as compared to the healthy, 4 and 8 week time points (n = 3 for each time point). (**K**) Elastin demonstrated a trend towards decreasing abundance at the 4 and 8 week time points as compared to the healthy and 2 week conditions.

Successful induction of MI in our rat model was confirmed through ventricular free wall thinning (Fig. 1A) and functional deterioration (Fig. 1B) following coronary artery ligation. The dense myocardium in the healthy heart was replaced over time by a thin, collagenous scar that was most evident at 8 weeks post-MI (Fig. 1A). Two-dimensional echocardiograms acquired along the long and short axis of the heart demonstrated both ventricular dilation and a loss of contractility following infarction (Fig. 1B). A significant decrease in ejection fraction (p < 0.001) was measured using Simpson’s 2D biplane method at 4 and 8 weeks post-MI relative to healthy non-infarcted controls (Fig. 1B). Successful decellularization of both healthy and infarcted hearts was demonstrated by a decrease in DNA content relative to the native myocardium (Supplementary Material and Figure S1). The scar region appeared distinctly more opaque than the non-infarcted region of the heart, and the scar size appeared to increase over time (Fig. 1C). Compositional analysis of the decellularized left ventricle of healthy hearts and scar of infarcted hearts did not demonstrate regional differences (p = 0.7313) in hydroxyproline content (HP), but did demonstrate differences across time points. At four (p = 0.002) and eight (p = 0.024) weeks post-MI, there was a significant increase in HP as compared to the healthy and 2 week post-MI groups (Fig. 2A).

Proteomics analysis using LC-MS/MS demonstrated an increase in the relative abundance of Collagen I during post-MI time points, but these data did not reach statistical significance (p = 0.108) (Supplementary Material and Fig. 2B). While the relative proportions of collagen III, V and VI did not significantly change over time, collagen IV increased in relative abundance post-MI, reaching a maximum at 2 weeks and steadily decreasing thereafter with significantly higher abundance than at 1 (p = 0.049), 4 (p = 0.046) and 8 weeks (p = 0.048) (Fig. 2D). Collagen XV was present at significantly higher proportions (p ≤ 0.041) in the healthy heart than at 1, 2, and 8 weeks post-MI (Fig. 2G). In addition to alterations in relative collagen content, periostin comprised a significantly greater proportion of the matrix at the 1 week time point as compared to the healthy (p = 0.019), week 4 (p = 0.022) and week 8 conditions (p = 0.008) (Fig. 2J). However, no significant changes in laminin and elastin content were detected following MI (p = 0.069 and p = 0.170, respectively) (Fig. 2). Collectively, proteomics analysis demonstrated that a variety of matrix components change (ie collagen IV, collagen XV and periostin) as a function of post-MI time. It is important to note that, LC-MS/MS analysis of the healthy and infarcted ECM did identify a number of basement membrane proteins (including fibulin-1 and nidogen) as well as proteins involved in the early formation of the ECM (emilin-1 and fibrillin-1) and in wound healing (fibrinogen), however all of these were either in very low abundance, did not change significantly during the remodeling process and/or do not contribute significantly to the mechanics of the ECM and thus we did not feel they were relevant to include here.

**Figure 3:**
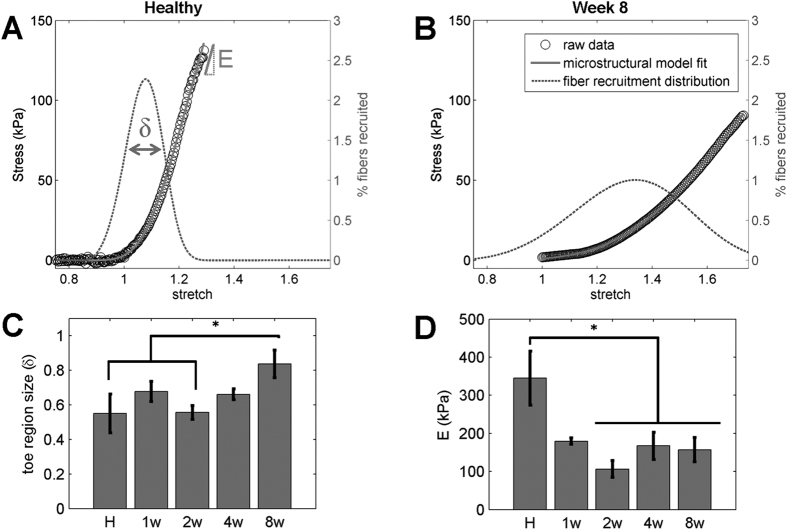
Changes in the mechanical response of the extracellular matrix following infarction. (**A**) A microstructural model of fiber recruitment was fit to the stress-stretch data of decellularized myocardium. The size of the toe region (δ) of the stress-stretch curve was defined by the width of the Weibull distribution of stretch values over which fibers were recruited and began to contribute stiffness, and elastic modulus (E) was defined by the slope of the stress-strain curve once all fibers have been recruited. (**B**) Decellularized infarcted hearts demonstrated a larger toe region and lower elastic modulus compared to healthy decellularized hearts in (**A**). (**C**) The toe region size was significantly greater at 8 weeks compared to 2 weeks and healthy tissue (p ≤ 0.0070). (**D**) The elastic modulus was significantly lower between 2–8 weeks relative to healthy tissue (p ≤ 0.0312). Significant differences (p < 0.05) identified through a two-way ANOVA among healthy (n = 6), week 1 (n = 5), week 2 (n = 9), week 4 (n = 8), and week 8 (n = 10) groups are denoted by *. No significant regional differences were identified.

Changes in matrix composition of the decellularized tissue also coincided with altered bulk tensile mechanical properties. Similar to the compositional measures, mechanical properties of the ECM varied with time (Fig. 3 and Table S1), but not as a function of location within the scar (p = 0.2121). However, regional differences did exist across the left ventricle of the infarcted heart when considering remote, border and infarct regions (Figure S2). Relative to the mechanical response of healthy ECM, infarcted decellularized tissue at week 8 exhibited an ECM with a reduced elastic modulus (Fig. 3A,B and Figure S3). In testing a subset of healthy and infarcted tissue, we found the ECM elastic modulus in the longitudinal direction was not significantly different from the circumferential direction (p = 0.2576) (Supplementary Material and Figure S4), and in fact, the moduli in these orthogonal directions were significantly correlated with each other (R = 0.859; p = 0.0132). This finding suggests that collagen fibers in decellularized tissue can freely rotate and bear similar loads in either direction. Consequently, all subsequent mechanical testing was performed in uniaxial tension in the circumferential direction to provide a means of characterizing the effective modulus of the collagen fibers in tension. A microstructural model with a Weibull distribution of stretch magnitudes at which fibers have rotated and begun to contribute stiffness proved a good fit (R^2^ = 0.9965 ± 0.0062) for the stress-stretch data spanning all time points. The size of the non-linear toe region of the stress-strain curve significantly increased following infarction, with week 8 post-MI toe regions significantly larger than that of healthy (p = 0.0026) and week 2 post-MI (p = 0.0070) tissues (Fig. 3C). Additionally, the ECM of healthy hearts exhibited significantly larger peak elastic moduli (E) than decellularized tissue at week 2 (p = 0.0022), week 4 (p = 0.0288), and week 8 (p = 0.0312) post-MI (Fig. 3D). These results demonstrate that although collagen content increases during scar remodeling, the infarct ECM has a reduced elastic modulus.

**Figure 4:**
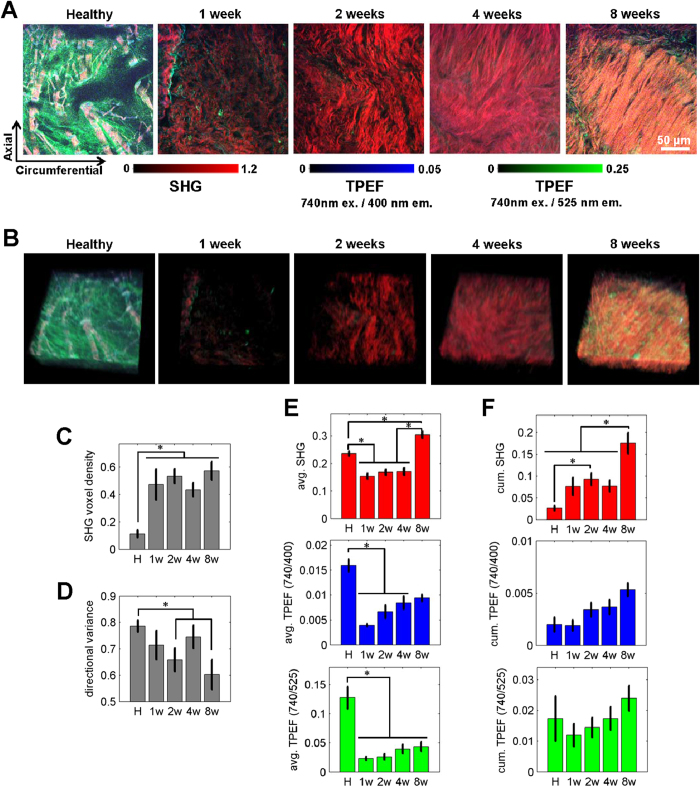
Changes in TPEF and SHG metrics of collagen following infarction. (**A**) Representative optical sections from an image volume of each decellularized sample type. (**B**) Corresponding 3D reconstructions of the image volumes for each sample. (**C**) The proportion of SHG-positive voxels increased following MI (p ≤ 0.0142). (**D**) The directional variance in the fiber orientation significantly decreased over time post-MI (p ≤ 0.0490). (**E**) The average SHG and TPEF intensities were computed within the SHG-positive voxels and exhibit a variety of differences between healthy and infarcted tissue. (**F**) The cumulative intensity from SHG-positive voxels was also computed and normalized by the total image volume, demonstrating an increase in total SHG intensity per volume upon infarction. Significant differences (p < 0.05) identified through a two-way ANOVA among healthy (n = 9), week 1 (n = 6), week 2 (n = 13), week 4 (n = 9), and week 8 (n = 6) groups are denoted by *. No significant regional differences were identified.

Healthy and infarcted hearts exhibited clear microstructural differences that were non-destructively characterized through multi-photon microscopy. Imaging metrics did not identify regional differences among regions within the infract scar, but regional differences across the remote, border and scar regions could be identified at 8 weeks post-MI (Figure S2). Qualitative examination of the image volumes revealed that decellularized healthy hearts are characterized by a sparse arrangement of relatively large diameter collagen fibers that are visible through SHG imaging, while the scar of infarcted samples consisted of a dense network of aligned, small diameter collagen fibers (Fig. 4A,B). A number of quantitative metrics can also be extracted from the images, once collagen regions are identified from SHG-positive voxels. Specifically, infarcted hearts contained a significantly higher density of SHG-positive (i.e. collagen containing) voxels at all post-MI time points compared to healthy hearts (p ≤ 0.0142) (Fig. 4C). These newly deposited collagen fibers of the scar region were more aligned with each other than the mature collagen fibers of the healthy tissue as quantified by the significantly lower fiber directional variance in the mean direction on week 2 (p = 0.0490) and week 8 (p = 0.0168) relative to healthy tissue (Fig. 4D).

In addition to changes in collagen organization, differences in SHG intensity were identified from the image volumes. The average SHG intensity within the collagen voxels differed among the healthy and post-infarct time points (Fig. 4E). The average SHG intensities during the first 4 weeks after infarction were significantly lower than healthy tissue (p ≤ 0.0014). However, at 8 weeks post-MI, the average SHG intensity was significantly greater than both healthy tissue (p = 0.0098) and tissue from earlier infarct time points (p < 0.0001). To obtain an optical estimate of the total collagen content within the tissue volumes, the average SHG intensity was integrated across all collagen voxels, and this cumulative SHG intensity was significantly increased in the week 2 post-MI samples when compared to the healthy ones (p = 0.0208). Cumulative SHG intensity continued to increase at later post-MI time points, and week 8 tissues were significantly greater than all other sample types (p ≤ 0.0215). Additional imaging experiments revealed SHG-based outcomes could be used to detect differences in remote, non-infarcted regions at late post-MI time points as well (Supplementary Material, Figure S2). The infarcted scar region demonstrated significantly higher SHG voxel density (p = 0.0002) and cumulative SHG intensity (p < 0.0001) compared to the remote regions (Figure S2). While we do not observe a significant difference between remote regions and healthy samples in terms of SHG voxel density, there is a trend of increasing SHG voxel density in the remote region indicating that with a higher number of samples and later time points our method may be sensitive to interstitial fibrosis that occurs with heart failure. Collectively, the increases in SHG intensity and fiber alignment over time provide distinct remodeling signatures within the scar and remote regions two months following MI.

Average TPEF intensities within the collagen fiber pixels were significantly correlated (p < 0.0001) among the three excitation-emission channels (Fig. 4, Supplementary Material, Table S2 and Figure S5), suggesting a similar sensitivity to collagen cross-linking status across a range of emission and excitation bands. Autofluorescent features were not restricted to the collagen containing regions, however, and a variety of ECM locations exhibiting no SHG intensity produced a strong TPEF signal in the healthy hearts (Fig. 4A,B). Collagen TPEF intensities within SHG-positive voxels for all three excitation-emission combinations were significantly lower at post-MI time points between weeks 1–4 relative to healthy hearts (p ≤ 0.0062) (Fig. 4E). However, a trend of increasing TPEF intensity during post-MI time points (Fig. 4E) resulted in significant differences between healthy and week 8 post-MI tissues for only the 525 nm emission channels (p ≤ 0.0106). Significant differences in TPEF intensity were noted between remote and infarcted regions after two months post-MI (p = 0.0019) but not between healthy and remote tissues (Figure S2). The reduced average TPEF intensity within the SHG-positive voxels of infarcted tissue relative to the healthy ECM suggests that newly deposited collagen within the scar does not become significantly cross-linked during the first 2 months after MI.

**Figure 5:**
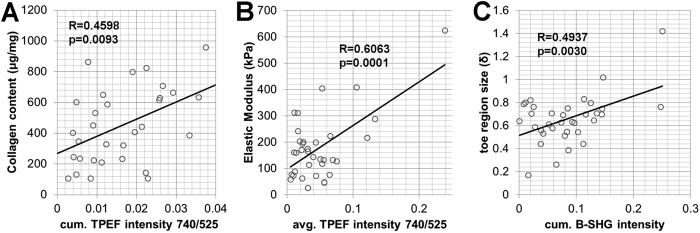
Correlations among collagen content, mechanical properties, and optical metrics in decellularized myocardium following infarction. (**A**) Collagen content was most strongly correlated (R = 0.4598, p = 0.0093, n = 33 samples) with the cumulative TPEF intensity (740 nm excitation and 525 nm emission) of the collagen containing voxels. (**B**) Elastic modulus was correlated (R = 0.6063, p = 0.0001, n = 34 samples) with average TPEF intensity within the collagen containing voxels. (**C**) The toe region size of the stress-stretch curve was correlated (R = 0.4937, p = 0.0030, n = 34 samples) with the cumulative SHG intensity.

Different quantitative metrics of collagen SHG and TPEF intensity were correlated with HP content and bulk mechanical properties (Table S3). HP content was not correlated with elastic modulus (R = −0.2085, p = 0.3281) or toe region size (R = 0.1462, p = 0.4954). HP content was, however, significantly correlated with cumulative intensity measurements of SHG and all TPEF channels (Table S3, p ≤ 0.0389), with the strongest correlation (R = 0.4598, p = 0.0093) between HP and the cumulative TPEF intensity at 740 nm excitation and 525 nm emission (Fig. 5A). The elastic modulus was significantly correlated with average TPEF intensities of the collagen-containing voxels (Table S3, p ≤ 0.0109), with the average TPEF intensity at 740 nm excitation and 525 nm emission yielding the strongest correlation (R = 0.6063; p = 0.0001) (Fig. 5C). Surprisingly, the elastic modulus was negatively correlated with collagen fiber voxel density (R = −0.4537, p = 0.0070) and cumulative SHG intensity (R = −0.3437, p = 0.0466), further indicating collagen content is not the primary determinant of ECM stiffness. The correlations among quantitative optical metrics, HP content and the ECM elastic modulus demonstrate that the substantial collagen deposited within the scar is not densely cross-linked and results in an ECM with a reduced elastic modulus.

## Discussion

This study evaluates the relationships among ECM mechanical properties, composition, and microstructure in the context of MI during scar remodeling through whole organ perfusion decellularization, non-linear optical microscopy, and tensile mechanical testing. Although a number of investigations have reported changes in the ECM and a stiffening of the infarcted myocardium post-MI^21^,^36^, those previous studies could not isolate the specific mechanical contribution of the ECM during infarct remodeling. Through decellularization, we have isolated the microstructural and material properties of the ECM and demonstrated that the matrix of the healthy heart is physically more mature with large, autofluorescent collagen fibers suggesting a high level of cross-linking (Fig. 4). Infarcted tissue, on the other hand, consists of a remodeled matrix that is functionally immature compared to its native form with an abundance of thin collagen fibers that lack cross-link fluorescence. The ECM of the post-MI scar contains more collagen (Fig. 2A) and more highly aligned fiber organization (Fig. 4A,D), but possesses a significantly lower elastic modulus than that of healthy tissue (Fig. 3D). It is not surprising that we detect a decrease in the bulk elastic modulus of the ECM upon the significant addition of immature collagen fibers that have a putatively lower modulus than that of the healthy, mature, collagen matrix. This significant deposition of new collagen fibers with lower elastic moduli is further highlighted by a negative correlation between elastic modulus and tissue thickness (R = −0.3805, p = 0.0184).

The decrease in bulk ECM modulus detected during scar remodeling in this study does not necessarily translate into a decrease in the overall elastic modulus of a native, cellularized myocardial wall. This is because the relative volume fraction of ECM to cellular constituents rapidly increases after MI as necrotic cells are cleared by inflammatory mediators and new collagen is deposited^22^. Relative to the ECM, the cellular constituents contribute minimally to the bulk myocardial stiffness during passive tension^37^; consequently, as the cellular density of the scar decreases drastically following MI, changes in the measured elastic modulus of the native, cellularized myocardium will be primarily driven by the decrease in volume occupied by cells. In fact, previous whole heart decellularization studies have demonstrated that cells account for a 15-fold greater proportion of the native myocardium thickness compared to the ECM, and this results in ECM elastic moduli values that are an order of magnitude greater than that of cellularized myocardium^23^. Through decellularization, our study provides a more nuanced understanding of how the composition, organization, and mechanical function of the isolated ECM change upon infarction, which complements previous biomechanical investigations of the native myocardium^7^,^15^,^38^.

Non-destructive multi-photon imaging of each specimen prior to loading identified a correlation between the elastic modulus of the ECM and the autofluorescence of the collagen fibers (Supplementary Material, Table S3 and Fig. 5B). Previous work has demonstrated that collagen autofluorescence is primarily associated with cross-linking status^29^,^30^,^31^,^39^, and groups have demonstrated that cross-linking plays a key role in determining fiber stiffness^16^,^29^,^33^. The newly deposited collagen matrix of the scar following MI is often void of collagen cross-links^40^ and lacks stiffness^16^,^36^,^40^, but the matrix matures with time as the number of collagen cross-links increases^15^. We observed similar trends in the decellularized infarct samples as measured by the average TPEF intensity within the collagen fiber voxels. Collagen autofluorescence emission measurements have historically been reported within the 400–500 nm emission range through one-photon excitation using 300–400 nm light^29^,^30^. However, TPEF measurements of collagen autofluorescence have previously demonstrated peak emission between 500–525 nm^24^, which is consistent with our findings (Fig. 4E). Elastin has also been speculated to contribute to ECM autofluorescence, but LC/MS-MS measurements of elastin do not match that of our TPEF intensity trends within the collagen regions (Figs 2K and 4). While our high-resolution imaging approach is capable of spatially discriminating elastin and collagen fibers, elastin autofluorescence may remain an important consideration for non-image-based spectroscopic approaches that seek to measure crosslinking. Although an association between fluorescence intensity and lysyl oxidase mediated cross-link density has been previously reported^31^, studies have also suggested contributions by non-enzymatic-mediated cross-links^39^. In fact, we have found an optical sensitivity to ribose-mediated cross-links with TPEF emission at 525 nm (Figure S6). It has been previously demonstrated that advanced glycation end-products (AGE) contribute to ECM remodeling during volume-overload heart failure^41^, but more research is needed to explore how they are altered as a function of time following infarction. In addition, it is important to identify how the different types of cross-links within collagen fibers contribute to changes in the measured autofluorescence intensity as the matrix matures and its stiffness increases (Fig. 4).

Similar to previous studies^40^, we detected an increase in collagen content during scar remodeling as measured through a hydroxyproline (HP) assay (Fig. 2A). Despite earlier reports which demonstrate an increase in collagen content as early as 1 week post-MI^36^, it is unclear whether that reported increase reflects new collagen deposition or a decrease in cellularity in the scar relative to the existing collagen matrix. By removing the cellular populations prior to analysis, our work demonstrates that significant collagen deposition is delayed. SHG images demonstrated an immediate increase in the volume fraction occupied by collagen following infarct (Fig. 4C), but this measurement was not correlated with HP content (Supplementary Material and Table S3), suggesting fibers at the early post-MI time points may be less densely packed with collagen fibrils. This assumption is supported by the significant correlation between the cumulative SHG intensity of the image volumes and HP content (Table S3). The intensity and directionality of the measured SHG signal can be altered by a number of factors, including fibril diameter, packing density, orientation, and inter-fibril spacing, which combine to influence the second-order nonlinear susceptibility at the focal spot and the phase matching conditions of the SHG process^39^,^42^,^43^. The cumulative intensity of the autofluorescence in SHG-containing voxels exhibited an even stronger correlation with HP content (Fig. 5A and Table S3) than SHG intensity. These findings suggest that possible changes in phase matching conditions may have produced variability in the SHG intensity that exceeded the variability in cross-link autofluorescence. Certainly additional work is necessary to fully elucidate the collagen fibril properties that drive changes in SHG intensity during scar remodeling.

Fourier analysis of SHG images revealed changes in collagen fiber orientation distributions as a function of post-infarct time point. As in previous work^44^, small diameter fibers within the scar were increasingly aligned with each other over time as demonstrated by the significantly lower directional variance (Fig. 4D). This lack of structural complexity within the remodeled matrix may be related to the significant decrease in collagen XV abundance in all post-MI time points. Previously, it has been demonstrated that *Col15a1−/−* mice possess thin and loose collagen bundles and a higher incidence of interstitial nonfibrillar protein aggregates within the myocardium relative to WT mice^45^. Therefore, the significant reduction in collagen XV following MI may contribute to the abundance of thin collagen fibers within the scar matrix due to a reduction in collagen XV-mediated collagen bundling^46^. Neither the mean fiber orientation, nor directional variance, was correlated with the elastic modulus. This lack of a correlation is likely due to the large, non-physiological rotations of collagen fibers that are possible within decellularized tissues during uniaxial loading. Additional mechanical testing revealed that the elastic moduli in the longitudinal and circumferential directions were not significantly different (Supplementary material, Figure S4), but rather strongly correlated (R = 0.8592; p = 0.0132), which further suggests that fibers are able to freely rotate in decellularized tissue and produce similar bulk stiffness values regardless of loading direction. Therefore, the modulus obtained in this study is likely a reflection of the modulus of an average collagen fiber aligned in the direction of loading, rather than that of a complex fiber network constrained by cellular constituents. With sensitivity to collagen fiber stiffness via uniaxial testing of decellularized tissue, we were able to identify a significant relationship between collagen cross-link autofluorescence and the ECM modulus. Future work utilizing microstructural models^47^,^48^,^49^, biaxial testing of fully-cellularized native tissue and our multi-photon microscopy outcomes will be critical in order to ascertain the relative contributions of fiber cross-linking and fiber alignment on the bulk mechanical properties of the myocardium during *in vivo* loading conditions. In particular, SHG imaging *during* loading^50^ may provide important insights into the relationships between ECM structure and mechanical function of the heart and lead to the development of much more sophisticated and biofidelic microstructural models. While sensitive to differences between the remote and scar regions of the infarcted heart, we were unable to distinguish regional differences among the isolated infarct scar sample location, which may have contained varying degrees of border zone ECM. The application of *in vivo* electromechanical mapping^51^, assessment of oxygen tension^52^ and/or characterization of regional cardiomyocyte apoptosis^53^ for the identification of the border zone could be utilized to understand the changing structure-function relationships of this specific ECM region during post-MI remodeling. In addition, the LC-MS/MS peptide reports did not detect several proteoglycans and glycosaminoglycans present in the cardiac ECM, which contribute significantly to the biophysical and biochemical properties of native tissue. The inability to detect these factors may be due to the decellularization process^54^ or the sensitivity of the LC-MS/MS to peptides with low abundance. Further optimization of these protocols, proposed testing of native tissues and/or immunostaining of cryosectioned tissues may help to identify the contributions of both proteoglycans and glycosaminoglycans to the changing structure-function relationships of the myocardial ECM during post-MI remodeling.

In summary, by eliminating the confounding effects of changing cellularity within the tissue through decellularization, we have uniquely identified structure-function relationships specific to the myocardial ECM. Changes in collagen autofluorescence intensity are correlated with the ECM elastic modulus and suggest sensitivity to collagen cross-linking density. This correlation between optical and mechanical characteristics of the ECM is in agreement with the results of a previous study, which identified correlations between multiphoton microscopy measures and mechanical properties during the decellularization of healthy, porcine cardiac tissue^55^. Here we significantly extend this work by carrying out a more robust characterization of the time-dependent remodeling of the ECM in infarcted tissue in order to identify specific changes to the structure-function relationship of the myocardial ECM during disease progression. Our findings highlight the importance of characterizing the ECM independently and non-destructively, which will be critical in order to assess the efficacy of clinical strategies that target the ECM remodeling process. Recently, several investigations have demonstrated how alterations in extracellular matrix remodeling following MI significantly impact function outcomes. For example, the targeted deletion of MMP-9 reduced enlargement and dilation of the left ventricle, which coincided with an increase in both collagen cross-linking activity and stiffness, but a reduction in collagen accumulation within the infarcted myocardium^56^. Alternative strategies have also manipulated proteoglycan expression and demonstrated that osteoglycin is a critical component for proper collagen fibrillogenesis during infarct remodeling. Osteoglycin null mice demonstrated a higher prevalence of mortality as a result of myocardial rupture due to impaired collagen network maturation within the scar^57^. These and other ECM-based therapies can benefit from the methodology described in the current study that provides an understanding of the relationships among the compositional, structural and functional properties of the myocardial ECM, which can otherwise be confounded by fluctuating cellular populations in the native heart. Additionally, the non-destructive, label-free, 3D imaging approaches used in this study have previously been applied *in vivo* for the characterization of native tissue^27^,^58^. Therefore, the multi-photon imaging metrics established here may have potential to serve as non-invasive biomarkers of ECM status to evaluate disease progression and therapeutic efficacy in preclinical and/or clinical research.

## Additional Information

**How to cite this article**: Quinn, K. P. *et al*. Optical metrics of the extracellular matrix predict compositional and mechanical changes after myocardial infarction. *Sci. Rep.* **6**, 35823; doi: 10.1038/srep35823 (2016).

**Publisher’s note**: Springer Nature remains neutral with regard to jurisdictional claims in published maps and institutional affiliations.

## Supplementary Material

Supplementary Information
