# Supplementary material for: Optical metrics of the extracellular matrix predict compositional and mechanical changes after myocardial infarction
Source: Sci Rep. 2016 Nov 7;6:35823. doi: 10.1038/srep35823 (PMC5098140; doi:10.1038/srep35823)
Supplement: Supplementary Information [file srep35823-s1.pdf]

Supplementary Material

Optical metrics of the extracellular matrix predict compositional and mechanical changes after myocardial infarction

Kyle P. Quinn[1,2*], Kelly E. Sullivan[1*], Zhiyi Liu[1], Zachary Ballard[1], Christos Siokatas[1,3], Irene Georgakoudi[1†], Lauren D. Black[1,4†]

[1]Department of Biomedical Engineering, Tufts University, Medford, MA, 02155, USA

[2]Department of Biomedical Engineering, University of Arkansas, Fayetteville, AR, 72701, USA

[3]Department of Chemistry, Aristotle University of Thessaloniki, Thessaloniki, 54124, Greece

[4]Cellular, Molecular, and Developmental Biology Program, Sackler School for Graduate Biomedical Sciences, Tufts University School of Medicine, Boston, MA 02111, USA

* Equal contribution

[†] **Correspondence should be addressed to:**
L.D.B. (lauren.black@tufts.edu) or I.G (irene.georgakoudi@tufts.edu)

Department of Biomedical Engineering
Tufts University
4 Colby Street
Medford, MA 02155
Fax: 617-627-3231
Phone: 617-627-4660

Supplementary Methods

Rat Model of Myocardial Infarction

All animal experiments were conducted in accordance with Tufts University guidelines and the US Animal Welfare Act. The animal protocol M2014-31 was approved by the Institutional Animal Care and Use Committee at Tufts University. *Myocardial infarction (*MI) was induced in male Sprague-Dawley rats that were 2 months of age or older and weighed between 250 and 275 grams. Animals were anesthetized with 4% isoflurane for induction and 2% for maintenance and anesthetic depth was monitored via respiratory rate and a toe pinch. Animals were intubated and the analgesic Bupivicaine was delivered subcutaneously at 2mg/kg in the intercostal region prior to the first incision. The heart was exposed with an incision between the fourth and fifth intercostal space. The left coronary artery was occluded with a 6-0 prolene suture. While the branching architecture of the artery varied from animal to animal, we ensured that blanching occurred across 40-50% of the left ventricular free wall. The analgesic Buprenorphine was delivered subcutaneously post-operatively at 0.05mg/kg post-operatively and dosed again every 12 hours for the next 72 hours following surgery. Animals with a significant infarct were allowed to recover for 1, 2, 4, or 8 weeks post-infarction. Healthy animals that did not undergo the surgical procedure served as controls. Animals were sacrificed via CO_2 asphyxiation and a thoracotomy. A total of 79 animals were used for the current study. For samples characterized through multiphoton microscopy, mechanical testing and hydroxyproline assessment, there was a minimum of 4 animals/condition.

Functional Assessment

Prior to sacrifice, animals were anesthesized with 4% isoflurane and the chest was shaved. A GE Vivid I ultrasound and a 12S-RS Phased Array Transducer (5.0 - 11.0 Mhz) were used to collect echocardiograms along the long and short axis of the heart. Ejection fraction was estimated using Simpson's 2D biplane method [1] (n=3 for each time point).

Infarct Matrix Isolation and Processing

Animals were sacrificed at the respective time points via CO_2 asphyxiation followed by a thoracotomy. As described previously[2], hearts were decellularized via retrograde perfusion with 1% sodium dodecyl sulfate (SDS). Briefly, the three branching points of the aorta were tied off with an ethicon suture and an 18G needle was advanced through the descending aorta. Hearts were removed from the chest cavity and perfused with 10 milliliters of phosphate buffered saline (PBS) followed by 2-4 liters of 1% SDS until both the scar and non-infarcted tissue became translucently clear and void of cellular material, which usually occurred within 48-72 hours. Hearts were subsequently rinsed with 0.5% triton-X for 12 hours and then rinsed with diH_20 for 72 hours (with water changed every 12 hours). Successful decellularization was confirmed through a Hoechst-based DNA assay of lyophilized ECM digested in Proteinase K for 24 hours at 55°C. Fluorescence intensity was normalized to that of the native, cellularized left ventricle. Whole hearts were photographed with a Nikon D3000 digital camera prior to, and

following decellularization. The infarcted region of the heart was excised and sectioned into three circumferential strips of similar width (between 2-4 mm) and identified as base, mid, or apex regions of the scar. For regional studies, additional 2-4 mm wide horizontal strips were isolated from the center of the scar, from the border zone and from the remote, non-infarcted myocardium following decellularization 8 weeks following MI. Samples were stored in 1x PBS at 4°C prior to imaging and mechanical testing.

Multi-photon Microscopy

Images of the decellularized tissue samples were obtained with a Leica TCS SP2 confocal microscope equipped with a tunable titanium-sapphire laser (Mai Tai; Spectra Physics; Mountain View, CA) and a water-immersion 63× objective (NA 1.2; 220 μm working distance). A field from both the epicardial and endocardial surfaces were randomly selected for imaging after placing the tissue on a glass coverslip (No. 1.5) in the center of the microscope stage. Samples were maintained in a humidified chamber to prevent dehydration during imaging. Images (512 × 512 pixels; 238 × 238 μm) were acquired at 5 μm z-steps (up to 40 slices) by two non-descanned photomultiplier tube (PMT) detectors using a filter cube containing a 700 nm short pass filter (ET700SP-2P), a 495 nm dichroic mirror (495DCXR), a 400(±10) nm emission filter (ET400/20X) placed before one PMT, and a 525(±25) nm emission filter (ET525/50M-2P) placed before the other PMT (Chroma; Bellows Falls, VT). Image volumes using both PMTs were acquired using 740nm and 800nm excitation wavelengths. For each image volume, contrast was optimized by adjusting PMT gain without saturating pixel intensity values. The 12-bit image intensities were normalized by PMT gain and laser power as previously described [3, 4].

Quantitative Image Analysis

The TPEF and SHG images were processed in Matlab to obtain quantitative metrics of the ECM microstructure. To define a collagen mask (i.e. the voxel locations within each acquired image containing collagen fibers), the SHG image was first filtered with a 5x5 Gaussian kernel (σ=1). To account for any offset of the background intensity value, the median intensity of the deepest slice (approximately 200μm from the surface) was subtracted from the filtered SHG image volume. The collagen mask included any voxel where the filtered SHG intensity exceeded 0.05. To minimize the effects of scattering, all subsequent analysis was performed on the portion of the image volume within the first 100μm from the surface (20 optical sections). The density of collagen-positive voxels within the imaged volume was computed for each volume, and the average intensity within the collagen mask was computed for each of the 4 image channels (SHG, TPEF 740/400, TPEF 740/525, TPEF 800/525). In addition, TPEF intensity divided by SHG intensity was computed to provide an alternative estimate of fluorescence per collagen molecule. To assess the cumulative collagen autofluorescence and SHG intensity within each image volume, the sum of all intensity values within the collagen voxels was computed and normalized by the total image volume.

Fiber orientation was quantified through methods similar to previous Fourier-based analysis approaches[5-7]. Each SHG image slice was apodized using a Hamming window, and then a two-dimensional (2D) power spectral density (PSD) map was obtained from each image through a discrete Fourier transform. The PSD value at each 2-D

(x,y) pixel location was summed in the z-direction. A polar coordinate system was assigned to each pixel location, with the center of the 2D PSD corresponding to the origin. Average PSD values were computed from pixel locations in discrete increments of 1°; pixel locations with radii (i.e. spatial frequencies) of less than 2.5 pixels or greater than 512 pixels were excluded from this computation. The mean fiber orientation and directional variance were computed from the average PSD value of each orientation from 0-180° [6]. To convert the mean fiber orientation into a linear metric, the absolute value of the difference between the mean fiber orientation and the longitudinal direction was computed for each sample.

Mechanical Testing

Prior to mechanical testing, the cross-sectional area of each decellularized tissue sample was calculated using a camera calibrated to measure thickness of the tissue sample and vernier calipers to measure width. Cyanoacrylate was used to mount the samples onto two footplates. The sample was then submerged in a bath of 1X PBS and the plates were carefully aligned in a custom-built uniaxial mechanical testing machine, described previously[8]. One foot was held in a fixed position, while the other foot was connected to a computer-controlled lever arm that measures displacement (1 μm resolution) and is outfitted with a force transducer (Model 400B, Aurora Scientific, Ontario, Canada; 0.3 mN resolution). Tissues were loaded in tension in the circumferential direction of the heart, which for these tissue sample locations corresponds to the direction of principal strain in vivo during maximum contraction[9].The undeformed length (L_0) of the tissue was defined at the lever position that corresponded to a 2kPa pre-stress, and tissue stretch (λ_t) was defined as ($d+ L_0)/L_0$, where d was the lever displacement. Samples were preconditioned with 6 cycles of quasi-static (45mm/min) displacement[10] to λ_t=1.54±0.08. Following 2 minutes of rest to allow for viscoelastic recovery in an unloaded configuration, the samples were loaded to λ_t =1.90±0.13 over 7 seconds, and force-displacement data were collected at 30 Hz.

Analysis of the Mechanical Response

First Piola-Kirchoff stress was computed from the force data during loading and the initial cross sectional area measurement. The data point corresponding to the initiation of mechanical failure was defined based on the first local maxima in the stress data exceeding 20 kPa; data points with stretch values exceeding the stretch at this local maximum stress value were removed from subsequent analysis.

Stress-stretch data were analyzed using a previously described probabilistic model of fiber recruitment for soft, connective tissues [11]. Tissue stress (σ_t) is modeled as a function of tissue stretch (λ_t),

$$\sigma_t(\lambda_t) = \int_{\gamma}^{\lambda_t} P_w(\lambda_s) \cdot [E \cdot (\lambda_t - \lambda_s)] d\lambda_s \,,$$

where λ_s defines the stretch at which a fiber begins to contribute to the elastic modulus (E), and γ defines the stretch value where the first fiber(s) contribute stiffness. P_w is the Weibull distribution of stretch values at which the tissue's fibers begin contributing stiffness as defined by,

$$P_w(\lambda_s) = \frac{\beta}{\delta}\left(\frac{\lambda_s - \gamma}{\delta}\right)^{\beta-1} e^{[(\frac{\lambda_s-\gamma}{\delta})^{\beta}]}, \ \lambda_s > \gamma$$

$$P_w(\lambda_s) = 0, \ \lambda_s \le \gamma,$$

where δ defines the x-scale of the distribution and β defines the shape of the Weibull distribution [11]. The scale of the distribution (δ) determines the size of the toe region of the stress-stretch curve. To prevent over-fitting of the stress-stretch curve, β was set to 4 following an initial assessment of fits with no parameter constraints. Additionally, the following constraints were imposed on the model: $\delta < 1.5$, and $0.5 \le \gamma < 1.5$, in order to ensure a majority of the fiber recruitment in the microstructural model occurred within the stretch values that were acquired experimentally. Ten randomized initial guesses within these constraints were generated, and the model was fit to the data using the lsqcurvefit function in Matlab. The parameters E and δ of the model fit with the smallest residual sum of squares for each curve were recorded. Model fits for samples where $\delta = 1.5$ (n=3) were removed from subsequent analysis.

Histological Assessment of Left Ventricular Free Wall Thinning

Additional hearts were isolated following CO_2 asphyxiation and were not decellularized, but characterized in their native state following fixation for 48 hours in 4% paraformaldehyde at 4°C. Hearts were rinsed in 1X PBS overnight followed by a 48 hour incubation in a 30% sucrose solution. Samples were embedded in an OCT solution and flash frozen in methyl butane solution immersed in liquid nitrogen. 20 µm heart sections were acquired with a Cryotome E Cryostat (Thermoscientific, Waltham, MA). Following removal of the OCT with 1X PBS, heart sections were stained with hematoxylin and eosin following standard protocols[12]. Images were acquired with a Leica DFC340 FX microscope (Wetzlar, Germany).

Collagen Content Assessment

Following mechanical testing and multi-photon imaging, decellularized samples were frozen in diH20 at -20°C overnight and then lyophilized for 24 hours. Dry tissue weight was measured and collagen content was characterized through a QuickZyme Total Collagen assay (QuickZyme Biosciences, Netherlands). Samples were hydrolyzed in 6M HCl at a concentration of 1mg/mL for 20 hours at 95°C. Following centrifugation at 13000g, the supernatant was analyzed for hydroxyproline content according to the manufacturer's instructions of the QuickZyme Total Collagen assay.

ECM Composition via Liquid Chromatography – Tandem Mass Spectroscopy (LC - MS/MS)

Additional samples were collected for proteomics analysis via LC-MS/MS. Following decellularization and lyophilization, samples were digested at a concentration of 5mg/mL in a solution consisting of 5M urea, 2M thiourea, 50 mM dithiothreitol and 0.1% SDS in PBS[13]. Samples were under constant agitation with a stir bar at 4°C. 72 hours later, protein was extracted through an acetone precipitation. Samples were analyzed though LC-MS/MS at the Beth Israel Deaconess Medical Center Mass Spectrometry Core Facility. The proportion of spectral counts for each matrix protein was calculated by taking the spectrum found for that protein and normalizing by the total number of spectra for structural ECM proteins (n=3 for each infarct time point) [14, 15].

Induction of glycation crosslinks in rat tail collagen fibers

The method used to induce sugar-derived (typically referred to as advanced glycation end product or AGE) crosslinks has been described in detail previously[16]. Briefly, tendon collagen fibers were isolated from rat tails and placed immediately in cold PBS. A portion of the collagen fibers (approximately 200 mg wet weight) were incubated in 4 ml PBS containing 0.2 M D-ribose and antibiotic at 35° Celsius for two weeks prior to TPEF imaging. Control fibers were incubated in PBS with antibiotic under the same conditions.

Supplementary References

1. Schiller NB, Shah PM, Crawford M, DeMaria A, Devereux R, Feigenbaum H, Gutgesell H, Reichek N, Sahn D, Schnittger I, et al. Recommendations for quantitation of the left ventricle by two-dimensional echocardiography. American Society of Echocardiography Committee on Standards, Subcommittee on Quantitation of Two-Dimensional Echocardiograms. *J Am Soc Echocardiogr* 1989;**2**:358-367.
2. Ott HC, Matthiesen TS, Goh SK, Black LD, Kren SM, Netoff TI, Taylor DA. Perfusion-decellularized matrix: using nature's platform to engineer a bioartificial heart. *Nat Med* 2008;**14**:213-221.
3. Quinn KP, Bellas E, Fourligas N, Lee K, Kaplan DL, Georgakoudi I. Characterization of metabolic changes associated with the functional development of 3D engineered tissues by non-invasive, dynamic measurement of individual cell redox ratios. *Biomaterials* 2012;**33**:5341-5348.
4. Quinn KP, Sridharan GV, Hayden RS, Kaplan DL, Lee K, Georgakoudi I. Quantitative metabolic imaging using endogenous fluorescence to detect stem cell differentiation. *Sci Rep* 2013;**3**:3432.
5. Sander EA, Barocas VH. Comparison of 2D fiber network orientation measurement methods. *J Biomed Mater Res A* 2009;**88**:322-331.
6. Quinn KP, Georgakoudi I. Rapid quantification of pixel-wise fiber orientation data in micrographs. *J Biomed Opt* 2013;**18**:046003.
7. Bayan C, Levitt JM, Miller E, Kaplan D, Georgakoudi I. Fully automated, quantitative, noninvasive assessment of collagen fiber content and organization in thick collagen gels. *J Appl Phys* 2009;**105**:102042.
8. Black LD, Brewer KK, Morris SM, Schreiber BM, Toselli P, Nugent MA, Suki B, Stone PJ. Effects of elastase on the mechanical and failure properties of engineered elastin-rich matrices. *J Appl Physiol* 2005;**98**:1434-1441.
9. Leitman M, Lysiansky M, Lysyansky P, Friedman Z, Tyomkin V, Fuchs T, Adam D, Krakover R, Vered Z. Circumferential and Longitudinal Strain in 3 Myocardial Layers in Normal Subjects and in Patients with Regional Left Ventricular Dysfunction. *J Am Soc Echocardiogr* 2010;**23**:64-70.
10. Jesudason R, Black L, Majumdar A, Stone P, Suki B. Differential effects of static and cyclic stretching during elastase digestion on the mechanical properties of extracellular matrices. *J Appl Physiol* 2007;**103**:803-811.
11. Hurschler C, Provenzano PP, Vanderby R, Jr. Application of a probabilistic microstructural model to determine reference length and toe-to-linear region transition in fibrous connective tissue. *J Biomech Eng* 2003;**125**:415-422.
12. Fischer AH, Jacobson KA, Rose J, Zeller R. Hematoxylin and eosin staining of tissue and cell sections. *CSH protocols* 2008;**2008**:pdb prot4986.
13. Ngoka LC. Sample prep for proteomics of breast cancer: proteomics and gene ontology reveal dramatic differences in protein solubilization preferences of radioimmunoprecipitation assay and urea lysis buffers. *Proteome Sci* 2008;**6**:30.
14. Naba A, Clauser KR, Lamar JM, Carr SA, Hynes RO. Extracellular matrix signatures of human mammary carcinoma identify novel metastasis promoters. *Elife* 2014;**3**:e01308.
15. Williams C, Quinn KP, Georgakoudi I, Black LD, 3rd. Young developmental age cardiac extracellular matrix promotes the expansion of neonatal cardiomyocytes in vitro. *Acta Biomater* 2014;**10**:194-204.
16. Tanaka S, Avigad G, Eikenberry EF, Brodsky B. Isolation and partial characterization of collagen chains dimerized by sugar-derived cross-links. *J Biol Chem*. 1988; **263**:17650-7.

Table S1. Summary of model parameters, maximum elastic modulus, elastic modulus at 1.25 stretch, and decellularized sample thickness.

	δ	γ	E(max)	E(1.25)	thickness (mm)
**Healthy**	0.55±0.28	0.68±0.14	345.01±173.62	86.55±44.05	1.14±0.19
**Week 1**	0.68±0.13	0.62±0.08	179.57±17.76	44.65±4.19	0.93±0.22
**Week 2**	0.56±0.12	0.60±0.09	106.43±65.71	26.65±16.56	1.74±0.44
**Week 4**	0.66±0.09	0.59±0.08	167.06±99.88	41.80±25.00	2.54±0.66
**Week 8**	0.85±0.24	0.53±0.05	152.29±104.24	37.99±26.20	1.51±0.53

Table S2. Correlations among multi-photon microscopy channels.

	SHG	TPEF 740/400	TPEF 740/525	TPEF 800/525
**SHG**	1	0. 4446	0. 3830	0. 4301
**TPEF (740/400)**	0.4446	1	0. 8622	0. 8769
**TPEF (740/525)**	0.3830	0.8622	1	0.9875
**TPEF (800/525)**	0.4301	0.8769	0.9875	1

Table S3. Correlations between optical metrics, collagen content, and mechanical parameters.

optical metrics		collagen content (μg/mg)	Size of the toe region (δ)	Peak elastic modulus (kPa)
**collagen fiber pixel density**		0.2609	0.2527	-0.4537*
**average intensity per collagen fiber pixel**	SHG	0.1548	0.3987*	0.0579
	TPEF (740/400)	-0.0381	-0.0812	0.4314*
	TPEF (740/525)	-0.0757	-0.3158	0.6063*†
	TPEF (800/525)	-0.0581	-0.3092	0.5755*
**cumulative intensity per image volume**	SHG	0.3728*	0.4937*†	-0.3437*
	TPEF (740/400)	0.3694*	0.2997	-0.1453
	TPEF (740/525)	0.4598*†	-0.0419	0.1920
	TPEF (800/525)	0.4314*	0.0303	0.0827

* p<0.05

† maximum correlation

Supplementary Figure Legends

Figure S1. Successful removal of cellular material following decellularization. Decellularization with 1% SDS reduces DNA content within the healthy left ventricle and scar of the infarcted hearts at each time point relative to the native cellularized left ventricle (n=3-4/time point).

Fig. S2. Comparisons between healthy samples (n=9), infarct scar regions (n=5), border regions (n=4) and non-infarct remote regions (n=3) of the myocardium 8 weeks post-MI. (A) Representative images of collagen SHG (red), TPEF at 525nm emission (green), and TPEF at 400nm emission (blue). Average values of (B) cumulative SHG intensity, (C) SHG voxel density, (D) directional variance, (E) average TPEF intensity at 525nm, (F) average TPEF intensity at 400nm and (G) elastic modulus indicate difference between healthy and infarct samples, as well as regional differences across the free wall of the infarcted left ventricle as measured by both imaging and mechanical testing. The infarcted regions possess greater collagen content than healthy samples, and either the remote or border regions as measured by cumulative SHG intensity (H vs. I, p<0.0001; R vs. I, p<0.0001; B vs. I, p <0.0001) and SHG voxel density (H vs. I, p<0.0001; R vs. I, p=0.0002; B vs. I, p=0.0005). In addition, the infarct region possesses a significant decrease in directional variance (H vs. I, p=0.0094; R vs. I, p=0.0130; B vs. I, p=0.0213) and TPEF intensity at 525 nm relative to the healthy samples (p<0.0001), and both the remote (p=0.0019) and border (p=0.0024). The elastic modulus of the decellularized ECM was significantly lower in the infarct region relative to the healthy samples (p=0.0161) and remote regions (p=0.0293).

Figure S3. Average stress-stretch curves for each group, based on the average microstructural parameters reported in Table S1.

Figure S4. Comparison of ECM mechanical properties in different loading directions. (A) Representative stress-stretch curves from healthy (n=4) and wk 1 post-MI (n=3) decellularized samples demonstrate that the elastic modulus within the linear region does not differ among loading directions. (B) No significant differences were detected in paired comparisons between circumferential and longitudinal loading directions (p=0.2576). Rather, the moduli in orthogonal directions were significantly correlated (R=0.8592; p=0.0132, n=7).

Figure S5. Correlation among TPEF channel intensities within SHG-positive voxels. (A) TPEF intensity at 400nm and 525nm emission wavelengths was correlated (R=0.8769, p<0.0001, n=43) at 740nm excitation. (B) TPEF intensities at 525nm emission were correlated between 740nm and 800nm excitation wavelengths (R=0.8622, p<0.0001, n=43). (C) TPEF intensity at 800nm/525nm was significantly correlated (R=0.9875, p<0.0001, n=43) with intensity at 740nm/400nm as well. Emission intensity in the 400nm channel was an order of magnitude weaker than the 525nm channels, contributing to the slightly lower correlation coefficients.

Figure S6. Comparison of TPEF intensities between collagen cultured in PBS (n=4) and ribose (n=2). Representative TPEF images of collagen cultured in PBS (A) and ribose (B) at 750nm excitation, 525nm emission. (C) A significantly higher level of average TPEF intensities was detected from the collagen cultured in ribose (p=0.044).

Figure S1.

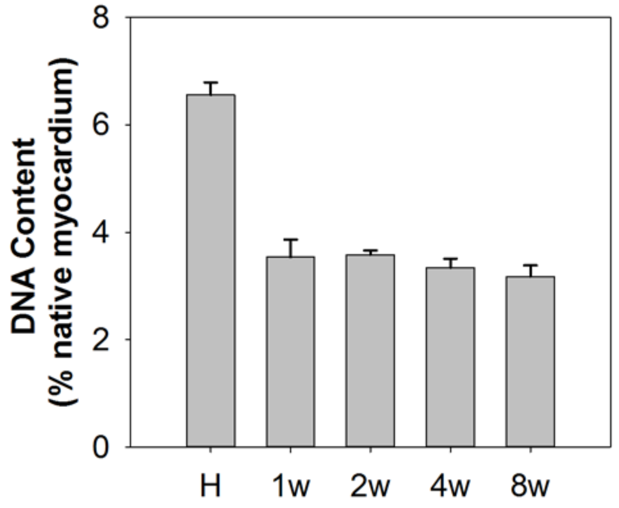

Figure S2.

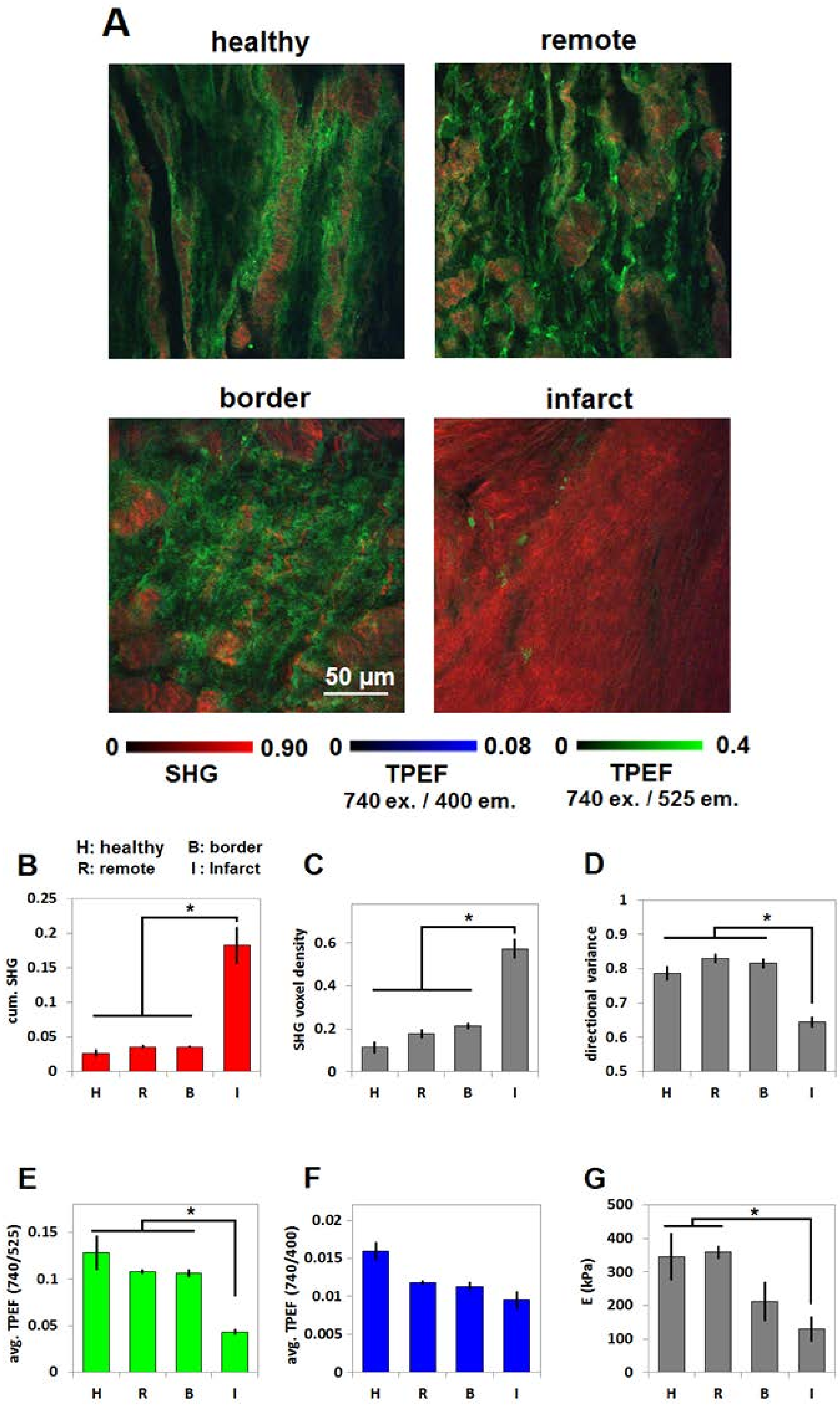
A
healthy
remote
border
infarct
50 µm
0
0.90
SHG
0
0.08
TPEF
740 ex. / 400 em.
0
0.4
TPEF
740 ex. / 525 em.
B
H: healthy
R: remote
B: border
I: Infarct
cum. SHG
C
SHG voxel density
D
directional variance
E
avg. TPEF (740/525)
F
avg. TPEF (740/400)
G
E (kPa)
H
R
B
I
*

Figure S3.

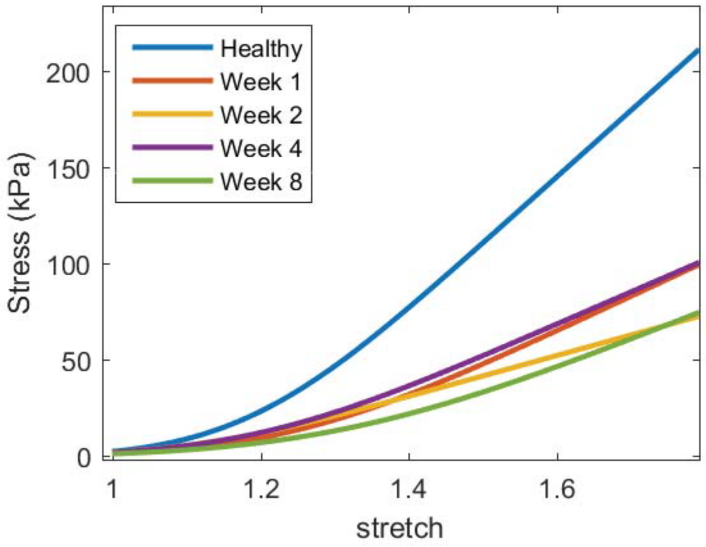
Healthy
Week 1
Week 2
Week 4
Week 8
Stress (kPa)
stretch
0
50
100
150
200
1
1.2
1.4
1.6

Figure S4.

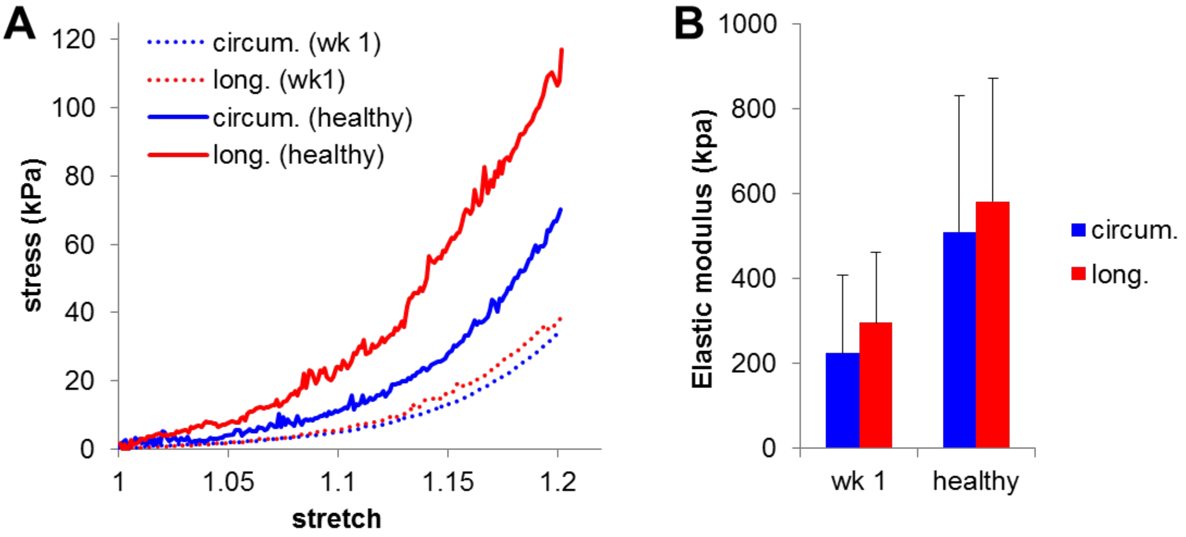
A
circum. (wk 1)
long. (wk1)
circum. (healthy)
long. (healthy)
stress (kPa)
0
20
40
60
80
100
120
1
1.05
1.1
1.15
1.2
stretch
B
Elastic modulus (kpa)
0
200
400
600
800
1000
wk 1
healthy
circum.
long.

Figure S5.

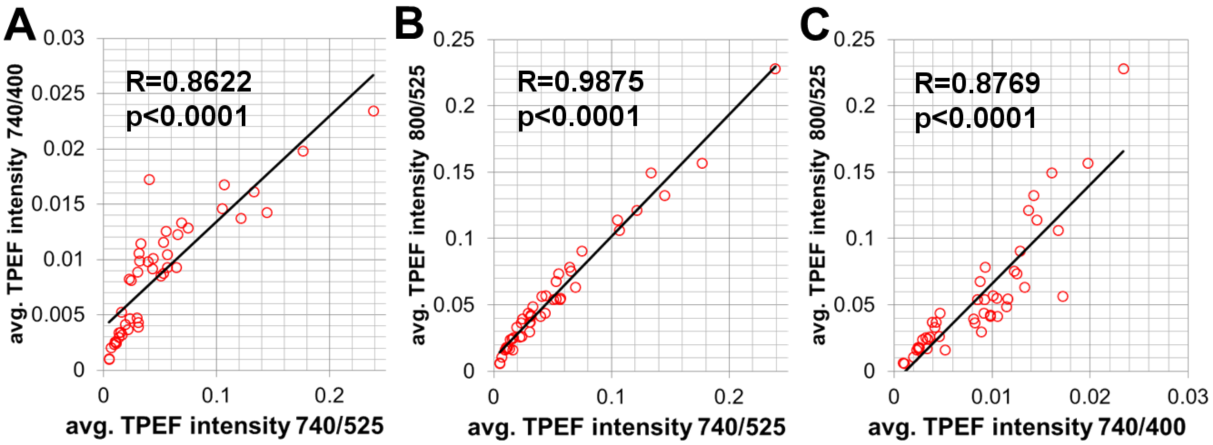
A
R=0.8622
p<0.0001
avg. TPEF intensity 740/400
avg. TPEF intensity 740/525
0.03
0.025
0.02
0.015
0.01
0.005
0
0
0.1
0.2
B
R=0.9875
p<0.0001
avg. TPEF intensity 800/525
avg. TPEF intensity 740/525
0.25
0.2
0.15
0.1
0.05
0
0
0.1
0.2
C
R=0.8769
p<0.0001
avg. TPEF intensity 800/525
avg. TPEF intensity 740/400
0.25
0.2
0.15
0.1
0.05
0
0
0.01
0.02
0.03

Figure S6.

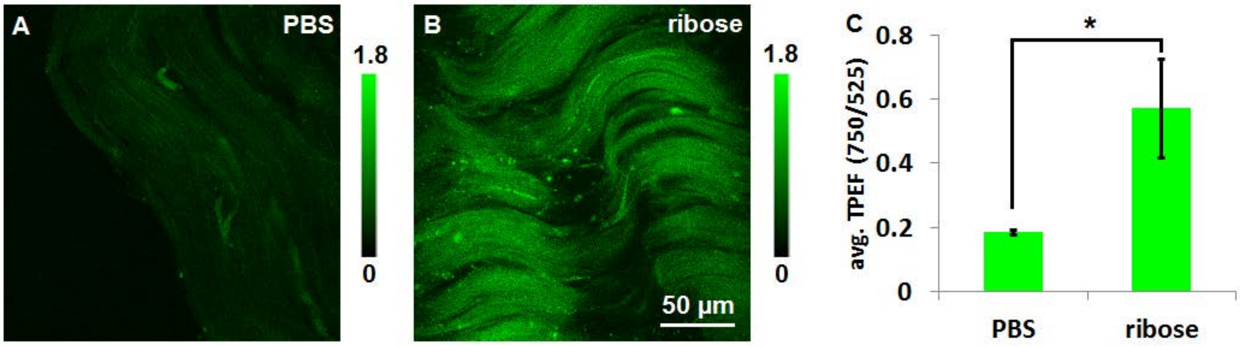
A
PBS
1.8
0
B
ribose
1.8
0
50 µm
C
*
avg. TPEF (750/525)
0.8
0.6
0.4
0.2
0
PBS
ribose
